# Comprehensive Characterization of the RNA Editomes in Cancer Development and Progression

**DOI:** 10.3389/fgene.2017.00230

**Published:** 2018-01-17

**Authors:** Haitao Luo, Shuangsang Fang, Liang Sun, Zhiyong Liu, Yi Zhao

**Affiliations:** ^1^Key Laboratory of Intelligent Information Processing, Institute of Computing Technology, Chinese Academy of Sciences, Beijing, China; ^2^University of Chinese Academy of Sciences, Beijing, China; ^3^Advanced Computer Research Center, Institute of Computing Technology, Chinese Academy of Sciences, Beijing, China

**Keywords:** post-transcriptional regulation, RNA editome, tumorigenesis, metastasis, transcriptome

## Abstract

RNA editing is a post-transcriptional event that leads to transcriptome diversity and has been shown to play important roles in tumorigenesis. However, dynamical changes and the functional significance of editing events during different cancer stages have not yet been characterized systematically. In this paper, we describe a comprehensive study of the RNA editome of four samples from different cancer stages for the same patient based on analysis of both whole-genome and transcriptome sequencing data. We identified 35,225 and 33,784 RNA editing events for poly(A)^+^ and poly(A)^-^ RNA sequencing data respectively in all four samples and show that 93 and 90% correspond to cancer stage-specific editing events. We also found that half of editing sites in 3′ UTR of coding genes were microRNA targets and most of the sites in the coding regions could lead to non-synonymous amino acid changes. Functional analysis of genes which suffered damaging non-synonymous editing events in each cancer stage show the gradual expansion of cancer related pathways accompanied by an increasing malignant grade of the samples. Our study, for the first time to our knowledge, comprehensively profiled and compared the editomes across the different cancer stages and revealed the functional impacts of RNA editing events during cancer development and progression.

## Introduction

RNA editing refers to programmed alterations in transcripts catalyzed by the double-stranded RNA-specific ADAR family of proteins. The emergence and rapid progress of high-throughput sequencing technology offers the promise of analysis of transcriptome-scale RNA editing events in cancer (Li et al., 2009; Peng et al., 2012; Kandoth et al., 2013; Baysal et al., 2017). Recently, several groups examined the high-throughput cancer RNA sequencing data to determine the frequency of RNA editing in various cancer types (Han et al., 2015; Paz-Yaacov et al., 2015; Zhang et al., 2016; Baysal et al., 2017). The results have suggested that RNA editing may promote tumorigenesis and metastasis by site-specific editing oncogenic genes, disrupting regulation by intronic or non-coding RNAs such as miRNAs.

Our previous study showed that transcriptomic analysis corroborated genetic alterations identified at the genomic level and could suggest the aggressiveness and metastatic potential of multifocal hepatocellular carcinoma (Miao et al., 2014). As the levels of RNA editing may constantly change during tumor development and progression, the functional impact of RNA editing on cancer will be different from those of permanent DNA mutations. It is therefore important that RNA editing studies should be carried out as a complement to genome sequence data to fully appreciate the full impact of nucleic acid sequence alterations in cancer (Baysal et al., 2017). Although RNA editing markedly increases the complexity of the cancer transcriptomes, cancer stage-specific recoding RNA editing events have not yet been comprehensively characterized.

In this study, we conducted a comprehensive study to characterize the RNA editomes in cancer development and progression based on the whole-genome and transcriptome sequencing data from four samples including: non-cancerous liver, primary liver tumor, an intrahepatic metastasis, and a portal vein tumor thrombus from the same patient and systematically analyzed dynamical changes and functional significance of editing events.

## Materials and Methods

### Sequencing Data

The sequencing data used in the current work were from our previous study (Miao et al., 2014). In brief, we sequenced the whole genomes of the DNA libraries for each sample using Illumina HiSeq^TM^ 2000 with 90-bp paired-end reads. Next, we fractionated total RNA into poly(A)^+^ RNA and poly(A)^-^ RNA and constructed whole-transcriptome sequencing libraries. We then performed strand-specific sequencing on both poly(A)^+^ and poly(A)^-^ RNA libraries with 90-bp paired-end reads. In total, an average of 97.8 Gb (31.6× coverage) genome and 7.6 Gb transcriptome sequences were obtained for each sample. The sequencing data were deposited at the European Genome-phenome Archive^1^, which is hosted by the EBI, under the accession number EGAS00001002338. Such deep sequencing data of each sample provides an ideal source for efficient characterization of the RNA editome.

### Illumina Reads Alignment

For both genome and transcriptom sequencing data, low-quality reads were removed if more than 10% of the bases were unknown or if more than 50% of the bases had a base quality lower than 5. The remaining high-quality paired-end reads were aligned to the NCBI Human Reference Genome Build 37 (hg19) using BWA (Li and Durbin, 2009) for genome sequencing data and using (Trapnell et al., 2009) for transcriptom sequencing data. The expression levels [Fragment Per Kilobase of exon model per Million mapped reads (FPKM)] of transcripts were calculated by Cufflinks (Trapnell et al., 2012).

### RNA Editing Sites Detection

Our bioinformatics analysis pipeline of RNA editing sites detection employed multiple steps and filters with stringent thresholds to facilitate unbiased detection of high reliable editing events. In brief, for each sample the variants were first identified from aligned reads using GATK (main parameters: -T UnifiedGenotyper -glm BOTH –standard_min_confidence_threshold_for_calling 2 –filter_reads_with_N_cigar -allowPotentiallyMisencodedQuals) (DePristo et al., 2011) and VarScan (main parameters: -min-var-freq 0.01) (Koboldt et al., 2009) software. For DNA variants, we combined the variants identified from GATK and VarScan for each sample. For RNA variants, the overlap variants between the two methods were used. Then, we focused on RNA–DNA variants only and sites in which DNA genotypes are the same as RNA genotypes and display more than one non-reference type were removed. The remaining variants were further filtered using the following criteria: the minimal sequencing quality score of SNV-corresponding nucleotide ≥ 20; reads covered depth ≥ 5; the minimal distance of a SNV site to its supporting reads’ ends ≥ 15; the minimal number of supporting reads ≥ 2. Finally, to eliminate germline variants, the remaining variants were cross-referenced against known SNP databases, including the 1000 Genomes Project^2^ and dbSNP (version 132) (Sherry et al., 2001).

### Functional Enrichment Analysis

We used DAVID (Database for Annotation, Visualization and Integrated Discovery) to enrich the Gene Ontology terms (Huang da et al., 2009). The *p*-value should be lower than 0.05. We then performed Enrichment Map analysis using Cytoscape to group and display the gene sets with similar functions (Merico et al., 2010). Statistically significant gene sets are linked if more than 80% of genes are shared by two gene sets.

## Results

### Identification of RNA Editing Events

**FIGURE 1:**
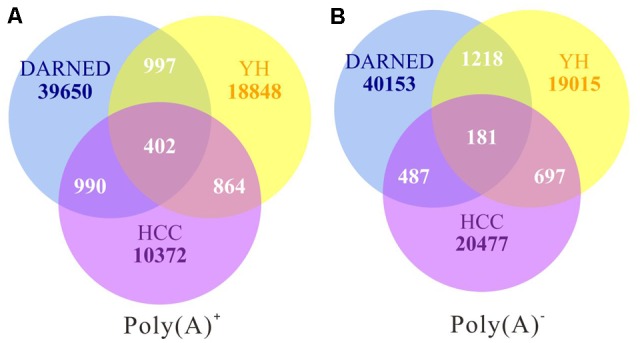
The overlap of RNA editing sites identified from poly(A)^+^ **(A)** and poly(A)^-^ **(B)** RNA sequencing data among different data sets. The data in the current study (HCC) were compared with those of DARNED and YH data. Numbers of sites that are unique or common among three data sets are shown.

To exhaustively analyze RNA editome during cancer development and progression, we first obtained whole-genome and transcriptome data from our previous study of four samples including: non-cancerous liver (NL), primary tumor (PT), an intrahepatic metastasis (IM), and a portal vein tumor thrombus (PTVV) from the same patient, who was a 49-year-old male diagnosed with a poorly-differentiated primary hepatocellular carcinoma (HCC) with multiple satellite lesions and PTVV (Miao et al., 2014). We used a similar bioinformatics analysis pipeline as previously described to identify editing sites for each sample (Peng et al., 2012; Ramaswami et al., 2012). After implementing multiple filters with stringent thresholds, a total of 45,505 and 40,432 sites were identified for poly(A)^+^ and poly(A)^-^ RNA-Seq data respectively in all four samples. Next, we cross-referenced this data against known protein-coding gene models in RefSeq (Pruitt et al., 2005) and long non-coding gene models in the Human Body Map lincRNA catalog (Djebali et al., 2012). This step shown that 22.6 and 16.5% of the identified sites for poly(A)^+^ and poly(A)^-^ data respectively were located in sequences that were either unannotated in the database or corresponded to overlapping transcripts on both strands. The remaining 35,225 and 33,784 sites for poly(A)^+^ and poly(A)^-^ data respectively were unambiguously mapped to known gene models, and thus were selected for further analysis. Then we compared A-to-I(G) editing sites with those in the DARNED database (Kiran and Baranov, 2010) and identified from the lymphoblastoid cell line of a male Han Chinese individual (YH) (Peng et al., 2012). The low overlap ratio among the three sets is consistence with previous study (Gommans et al., 2009; Peng et al., 2012) and that suggested the variety and diversity of post-transcriptional modification due to low evolutionary cost (**Figure 1**).

### Cancer Stage Related Editing Events

**FIGURE 2:**
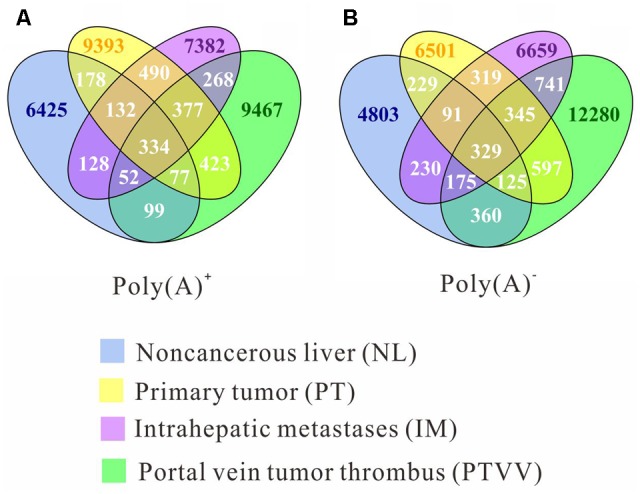
The overlap of RNA editing sites among four cancer stages: non-cancerous liver (NL, blue), primary tumor (PT, yellow), an intrahepatic metastasis (IM, purple), and a portal vein tumor thrombus (PTVV, green), which were identified from poly(A)^+^ **(A)** and poly(A)^-^ **(B)** RNA sequencing data. The numbers of sites that are unique or common among four samples are shown.

**FIGURE 3:**
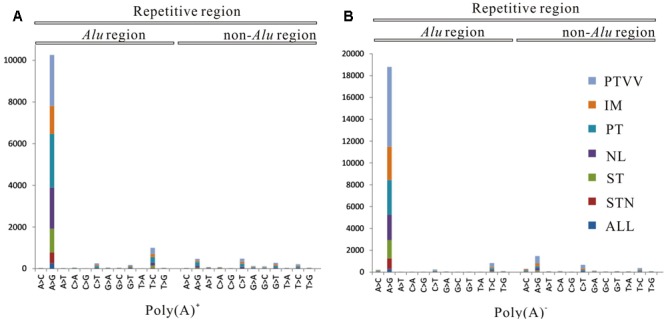
Distribution of 12 types of RNA editing sites identified from poly(A)^+^ RNAs **(A)** and poly(A)^-^ RNAs **(B)** in *Alu* and repetitive non-*Alu* regions. Seven editing patterns: omnipresent across all four samples (ALL), partially shared by both tumourous and non-cancerous liver samples (STN), partially shared by only tumourous samples (ST), non-cancerous liver (NL), primary tumor (PT), an intrahepatic metastasis (IM), and a portal vein tumor thrombus (PTVV), are shown as different colors.

To systematically analyze RNA editomes during cancer development and progression, we divided the editing sites into seven patterns: omnipresent across all four samples (ALL); partially shared by both tumourous and non-cancerous liver samples (STN); partially shared by only tumourous samples (ST); remaining four patterns are sites unique to each sample (NL, PT, IM, PTVV). Distribution of editing events for both poly(A)^+^ and poly(A)^-^ data across each pattern are displayed (**Figure 2**). The results show that 93 and 90% sites correspond to stage specific editing events. Next, we investigated the editing events in *Alu* and repetitive non-*Alu* regions (**Figure 3**). Consistent with the previous observations, A-to-G editing events was significantly enriched in repetitive *Alu* elements when compared with the other 11 types of variants. Taken together with the above results, during cancer development and progression, only a few editing sites were preserved across different stages. Most were stage specific events and that strongly suggests the distinct adaptiveness of editome in cancer evolutionary process.

### Analysis of the Editing Sites across Gene Region

**FIGURE 4:**
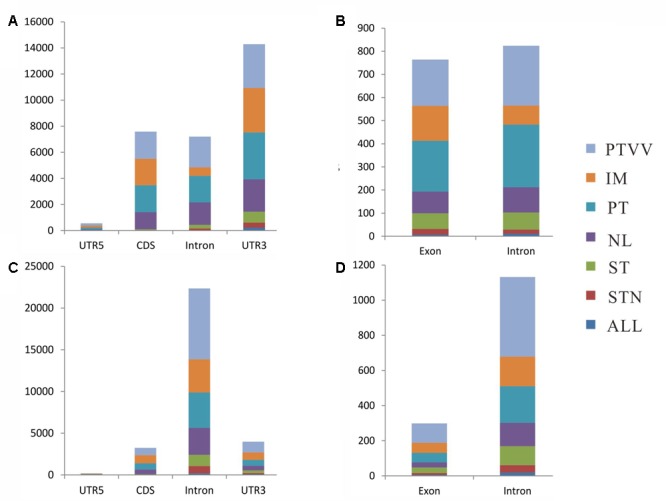
Distribution of RNA editing sites identified from poly(A)^+^ RNAs **(A,B)** and poly(A)^-^ RNAs **(C,D)** in different regions of genes. For protein-coding genes, the distribution of the number of editing sites in 5′-untranslated region (UTR5), CDS, Intron, and UTR3 are shown in **(A,C)**. For long non-coding genes, the distribution of the number of editing sites in exon and intron are shown in **(B,D)**. Seven editing patterns are shown as different colors as described in **Figure 3**.

We next studied the location attributes of the identified RNA editing events in each stage of cancer development and progression (**Figure 4**). Among sites in poly(A)^+^ RNA, most of those were located in 3′-untranslated region (UTR) of coding transcripts. In comparison, the equivalent editing sites of exons and introns were observed in long non-coding transcripts. Interestingly, almost all the conserved sites across four cancer stages were located in non-coding regions, particularly in 3′ UTR of coding genes, and less than 4% conserved sites were in coding regions (CDS). However, further analysis of the sites in poly(A)^-^ RNAs showed distinct location attributes although conserved editing sites were also enriched in non-coding regions, of which editing events in the introns is predominant irrespective of coding or non-coding transcripts. Moreover, the portion of editing sites in the exons of non-coding genes is decreased in poly(A)^-^ RNAs when compared with that in poly(A)^+^ RNAs.

**FIGURE 5:**
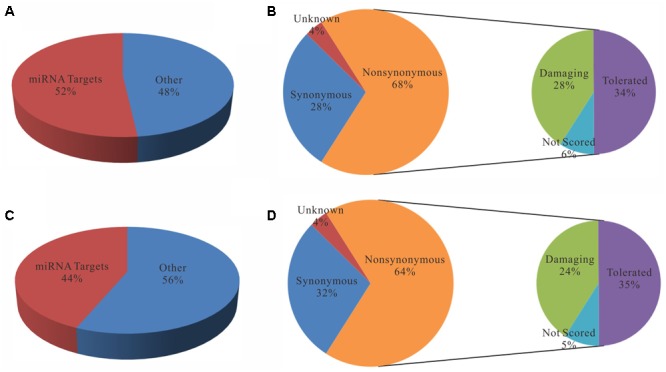
Statistics of RNA editing sites located in 3′-untranslated regions **(A,C)** and CDS regions **(B,D)**. **(A,C)** Pie charts display the proportions of sites that are located in miRNA target regions. **(B,D)** Pie charts display the proportions of sites with different functional effects predicted by SIFT annotation (Kumar et al., 2009).

**FIGURE 6:**
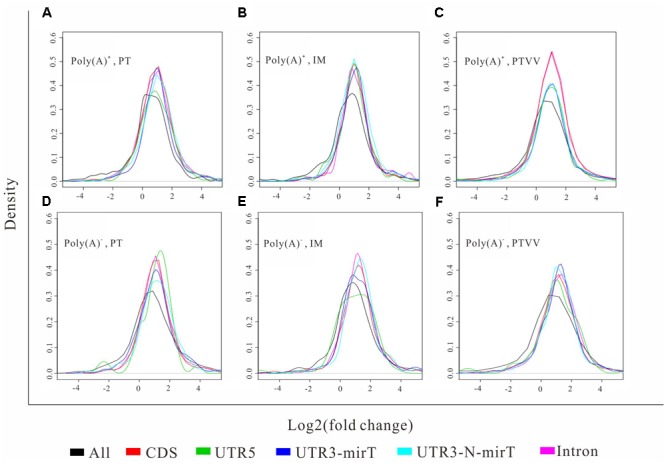
Shows the distribution of expression changes of transcripts with different editing events. The expression changes of transcripts that were edited in the primary tumor (PT) **(A,D)**, intrahepatic metastases (IM) **(B,E)** or portal vein tumor thrombus (PTVV) **(C,F)** separately but not in the non-cancerous liver are evaluated and plotted based on both poly(A)^+^ (up panel) and poly(A)^-^ RNA (bottom panel) sequencing data. The transcripts with different editing events, including editing events occurred in UTR5, CDS, miRNA targets in UTR3, non-miRNA targets in UTR3, and Intron regions, and all transcripts as background were analyzed. Statistical significance of each editing event versus background was calculated by the Kolmogorov–Smirnov test.

To further evaluate the functional consequence of editing events, we analyzed the sites in 3′ UTR and coding regions as well as editing impacts on gene expression. First, we found that the editing sites in 3′ UTR were significantly enriched in microRNA targets (*p-*value ∼ 0, hypergeometric test). Specifically, 52 and 44% editing sites in 3′ UTR were microRNA targets which predicted by miRTar (Hsu et al., 2011) for poly(A)^+^ and poly(A)^-^ RNAs respectively (**Figure 5**). The top 10 miRNAs according to the number of edited targets were shown in **Table 1**. In addition, more than half of the sites in coding regions could led to non-synonymous amino acid changes (**Figure 5**). Then we compared these editing genes with those identified in previous study (Paz-Yaacov et al., 2015). The results shown that 95% of the genes that were edited in microRNA targets or occurred non-synonymous changes in liver cancer as described in previous work are reported in our study. Next, to determine whether editing events have any impact on gene expression, we evaluated expression changes of transcripts that were edited in the primary tumor, intrahepatic metastases or portal vein tumor thrombus separately but not in the non-cancerous liver. Unexpectedly, although RNA editing sites in different gene regions may have an impact on gene function via microRNA pathway or non-synonymous changes, there was little effect on gene expressions as shown in current study (**Figure 6**).

**Table 1:** The top 10 miRNAs according to the number of edited targets for poly(A)^+^ RNAs and poly(A)^-^ RNAs data.

	poly(A)^+^ RNAs	poly(A)^-^ RNAs
1	hsa-miR-30b^∗^	hsa-miR-1827
2	hsa-miR-1827	hsa-miR-30b^∗^
3	hsa-miR-612	hsa-miR-1287
4	hsa-miR-4266	hsa-miR-20a
5	hsa-miR-485-5p	hsa-miR-20b
6	hsa-miR-1287	hsa-miR-17
7	hsa-miR-3188	hsa-miR-106a
8	hsa-miR-339-5p	hsa-miR-3163
9	hsa-miR-541	hsa-miR-93
10	hsa-miR-298	hsa-miR-548c-3p

*^∗^Denote that the miRNA is from the opposite arm of the precursor.*

### Functional Analysis of Editing Genes in Cancer Development and Progression

**FIGURE 7:**
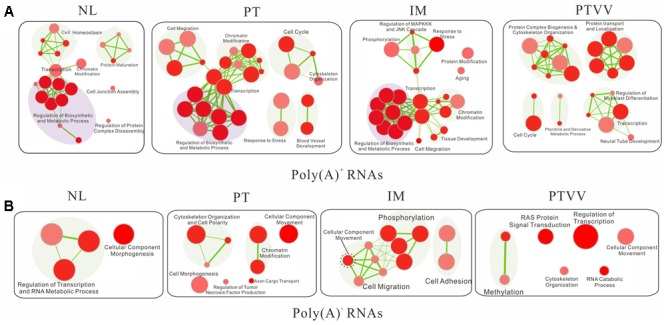
Functional enrichment maps of the protein-coding genes that suffered damaging non-synonymous editing events based on both poly(A)^+^ **(A)** and poly(A)^-^ RNA **(B)** sequencing data. For each sample, each node represents a gene set; the size of the node is indicative of the number of genes and the color intensity reflects the level of significance. The thickness of each line is proportional to the number of genes shared by connected gene sets.

To characterize the biological significant of stage-specific editomes in cancer development and progression, we tested for functional enrichment of gene sets which suffered damaging non-synonymous editing events in each sample. Among damaging non-synonymous variants of poly(A)^+^ RNA (**Supplementary Data Sheet S1**), although there were distinct editing profiles in different stages of cancer, similar biological function impacts were observed. Furthermore, the gradual expansion of cancer related pathways was clearly shown with the increasing malignant grade of samples (**Figure 7A**). Specifically, there were no cancer pathways presented in non-cancerous liver, and several malignant processes arise in primary tumor including cell migration, cell cycle and blood vessel development. In addition, the regulation of MAPKKK and JNK cascades, which were connected to responses to stress and phosphorylation modules in enrichment maps and play important roles in cell death, appeared in intrahepatic metastases. In the portal vein tumor thrombus, the predominant functional impacts of editing events occurred at the post-translational level, such as protein complex biogenesis and protein localization. Furthermore, regulation of biosynthetic and metabolic process modules was commonly deregulated in all intrahepatic samples but not in the portal vein tumor thrombus and that was consistent with the special metabolic function of liver tissue.

Further analysis of the damaging non-synonymous sites in poly(A)^-^ RNAs (**Supplementary Data Sheet S2**) showed that they have a similar, but still have exclusive functional impacts compared with those in the poly(A)^+^ RNAs (**Figure 7B**). Consistent with above findings, the cell migration module also presented in the primary and metastatic tumors, from cellular component movement expanding to cell migration during tumor metastasis. Moreover, two modules, including regulation tumor necrosis factor production and Ras protein signal transduction module, which have important roles in cancer, were unique to the primary tumor and portal vein tumor thrombus respectively. Notably, differing poly(A)^+^ RNAs, biological synthesis and metabolism process were not present in all intrahepatic samples of poly(A)^-^ RNAs and that may imply the different regulatory mechanism between the two types of RNAs. Taken together, editing events in both poly(A)^+^ and poly(A)^-^ RNAs may have great impacts in cancer development and progression.

## Discussion

Initial studies of RNA editing in cancer suggests that both genome and transcriptome sequencing experiments are required to capture all the editing events comprehensively. In the current study, we conducted a systematical analysis of cancer RNA editomes based on the whole-genome and transcriptome sequencing data from four samples including: non-cancerous liver, primary tumor, an intrahepatic metastasis and a portal vein tumor thrombus from the same patient, which represented the whole process of tumorigenesis and metastasis.

According to the presence or absence of a poly(A) tail at 3′ ends, RNAs can be physically classified into poly(A)^+^ or poly(A)^-^ transcripts (Yang et al., 2011). Most studies focused on poly(A)^+^ transcripts and relatively little is known about poly(A)^-^ transcripts, which is a significant portion of transcripts. Previous studies revealed that many mRNAs may lack poly(A) tails and such types of mRNA are overrepresented in specific functional categories (Yang et al., 2011). In this study, we used deep sequencing data to explore the repertoire of both poly(A)^+^ and poly(A)^-^ RNAs from four stages of cancer samples, and derived a comprehensive RNA editing landscape.

Our work not only focused on the editing sites in coding transcripts, but also in long non-coding transcripts, which have emerged as a new class of functional RNAs and play important roles in cancer (Gupta et al., 2010; Liao et al., 2011). The latest version of the NONCODE database has collected more than 30,000 human and 20,000 mice long non-coding RNAs, many of which were annotated as cancer related functions (Luo et al., 2016; Zhao et al., 2016). The higher proportion of sites in the exons of poly(A)^+^ non-coding genes compared with poly(A)^-^ ones, suggesting that more functional elements are related to editing events enriched in these regions.

Although nearly half of editing sites in 3′ UTR were microRNA targets or could led to non-synonymous amino acid changes, there were little effect on gene expressions. The results suggested RNA editing could occur via different mechanisms to influence gene function, but it may just one complex systems of gene regulation during post-transcriptional processes.

In the functional enrichment study, we found distinct editing sites and accordant functional impacts across each stage of tumorigenesis and metastasis, which may reflect the pattern of implement function in the RNA editome. Our study suggested that the dysregulation of RNA editing events may contribute to the altered transcriptional program necessary to sustain carcinogenesis, and may lead to identification of novel diagnostic and prognostic markers in cancer.

## Author Contributions

HL, ZL, and YZ designed the study. HL, SF, and LS performed analyses. All authors contributed to writing the manuscript and approved its final version.

## Conflict of Interest Statement

The authors declare that the research was conducted in the absence of any commercial or financial relationships that could be construed as a potential conflict of interest.

## Footnotes

1 https://www.ebi.ac.uk/ega/

2 http://www.1000genomes.org/

## Supplementary Material

DATA SHEET S1 The list and detailed description of damaging non-synonymous variants of poly(A)^+^ RNA.

DATA SHEET S2 The list and detailed description of damaging non-synonymous variants of poly(A)^-^ RNA.
